# The survival decrease in gastric cancer is associated with the methylation of B-cell CLL/lymphoma 6 member B promoter

**DOI:** 10.1098/rsob.140067

**Published:** 2014-07-09

**Authors:** Jingyu Deng, Han Liang, Qiuping Dong, Yachao Hou, Xingming Xie, Jun Yu, Daiming Fan, Xishan Hao

**Affiliations:** 1 Department of Gastroenterology, Tianjin Medical University Cancer Hospital, City Key Laboratory of Tianjin Cancer Center and National Clinical Research Center for Cancer, Tianjin, People's Republic of China; 2 Central Laboratory, Tianjin Medical University Cancer Hospital, City Key Laboratory of Tianjin Cancer Center and National Clinical Research Center for Cancer, Tianjin, People's Republic of China; 3 Institute of Digestive Disease, Li Ka Shing Institute of Health Science, Chinese University of HongKong, Shatin, Hong Kong; 4 State Key Laboratory of Cancer Biology and Institute of Digestive Diseases, Xijing Hospital, Fourth Military Medical University, Xi'an, People's Republic of China

**Keywords:** stomach, neoplasm, B-cell CLL/lymphoma 6 member B, survival, methylation

## Abstract

The methylation of B-cell CLL/lymphoma 6 member B (BCL6B) DNA promoter was detected in several malignancies. Here, we quantitatively detect the methylated status of CpG sites of BCL6B DNA promoter of 459 patients with gastric cancer (GC) by using bisulfite gene sequencing. We show that patients with three or more methylated CpG sites in the BCL6B promoter were significantly associated with poor survival. Furthermore, by using the Akaike information criterion value calculation, we show that the methylated count of BCL6B promoter was identified to be the optimal prognostic predictor of GC patients.

## 2. Introduction

Gastric cancer is the third most common cancer in China, with an incidence rate 2–3 times higher than the global average. This rate, combined with the large population, means that gastric cancer in China accounts for more than 40% of new gastric cancer cases world wide [1]. Owing to the lack of highly specific biomarkers of carcinogenesis and the precisely prognostic predictors of GC, the overall survival (OS) of patients has not significantly improved [2]. B-cell CLL/lymphoma 6 member B (BCL6B), also known as BAZF, is a member of the proto-oncogene Bcl6 family genes that encodes a sequence-specific transcriptional repressor containing the BTB/POZ domain in NH2-terminal region and zinc finger motifs in COOH-terminal region [3]. The expression of BCL6B mRNA was ubiquitously detected in many kinds of human tissues. The expression patterns of BAZF mRNA suggest that BCL6B may regulate differentiation in stages or lineages [4]. The 17-amino-acid region in the middle portion of BCL6 is a functional domain of transcriptional repressor activity and is responsible for inducibility of apoptosis in NIH3T3 cells [5]. BCL6b is also identified to be required for the enhanced magnitude of the secondary response of memory CD8^+^ T cells [6]. Recently, researchers reported that BCL6B played a pivotal role as a potential tumour suppressor in GC, and the detection of methylation of the BCL6B DNA promoter might be deemed an independent biomarker for the prognosis of GC [7]. Hypermethylation of the CpG islands in BCL6B promoter was demonstrated to be detected in 42.5% plasma DNA samples from GC patients, while no methylation of BCL6B promoter was found in the plasma DNA of healthy controls [8].

In view of the small number of patients and the qualitative method of the promoter methylation in those studies, we intend to detect the quantitative methylated levels of BCL6B DNA promoter in a large-scale patient study for elaborate elucidation of the prognostic predicted value of BCL6B promoter methylation in GC.

## 3. Results

### 3.1. Patient demographics

All 459 GC patient clinicopathological characteristics are listed in table 1. The median OS of all patients was 21 months. Of 459 patients, 61 (13.26%) were alive when the follow-up was over.

**Table 1.** Patient information in this study.

gender	
male	314 (68.41%)
female	145 (31.59%)
age at surgery	
≤60	270 (58.82%)
>60	189 (41.18%)
tumour size	
<4.0	66 (14.38%)
≥4.0	393 (85.62%)
tumour location	
upper third	113 (24.62%)
middle third	117 (25.49%)
lower third	201 (43.79%)
more than 2/3 stomach	28 (6.10%)
depth of tumour invasion (T stage)	
T1	5 (1.09%)
T2	46 (10.02%)
T3	284 (61.87%)
T4	124 (27.02%)
number of metastatic lymph nodes (N stage)	
N0	110 (23.97%)
N1	164 (35.72%)
N2	107 (23.31%)
N3	78 (17.00%)
location of lymph node metastasis	
no	110 (23.97%)
perigastric	159 (34.64%)
extragastric	190 (41.39%)
Lauren classification	
intestinal	122 (26.57%)
diffuse	319 (69.50%)
mixed	18 (3.93%)
methylated CpG site count	
2 or less	220 (47.93%)
3 or more	239 (52.07%)
methylated status of CpG +79	
unmethylated	229 (49.89%)
methylated	230 (50.11%)

### 3.2. Protein and mRNA expression of B-cell CLL/lymphoma 6 member B in gastric cancer tissues and normal gastric mucosal tissues

**Figure 1.**
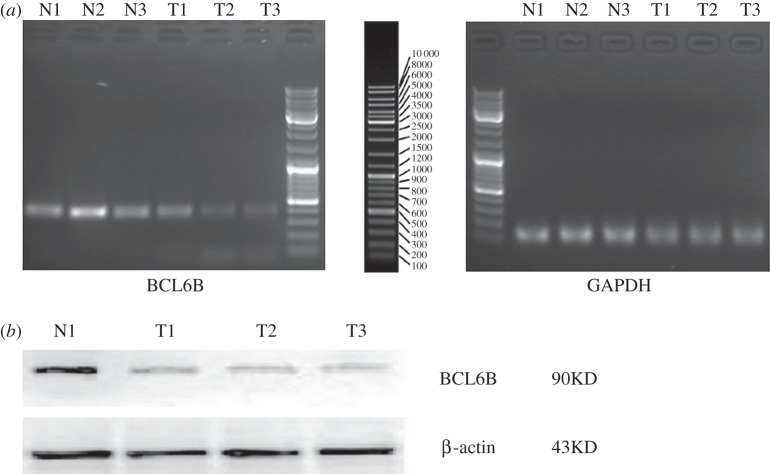
(*a*) BCL6B mRNA expression (RT-PCR) in GC tissues and in normal gastric mucosal tissues. (*b*) Western Blot analysis for BCL6B protein expression in GC tissues and in normal gastric mucosal tissues. T, GC tissues; N, normal gastric mucosal tissues.

BCL6B mRNA expression was detected in 25 of 459 GC tissues and 25 normal gastric mucosal tissues by reverse transcription polymerase chain reaction (RT-PCR; figure 1*a*). We also found that there were significant differences of BCL6B mRNA expression among 25 GC tissues. The mean value of relative mRNA expression of BCL6B in 25 GC tissues was 0.410 ± 0.118, whereas the mean value of relative mRNA expression of BCL6B in 25 normal gastric mucosal tissues was 1.561 ± 0.406. The mean value of relative mRNA expression of BCL6B in 25 GC tissues was lower than that in 25 normal gastric mucosal tissues (*p* = 0.016).

Similarly, BCL6B protein expression was also detected in 25 of 459 GC tissues and 25 normal gastric mucosal tissues by Western blot, simultaneously (figure 1*b*). We found there were significant differences of BCL6B protein expression among 25 GC tissues. The mean value of relative protein expression of BCL6B in 25 GC tissues was 0.753 ± 0.144, whereas the mean value of relative protein expression of BCL6B in 25 normal gastric mucosal tissues was 1.662 ± 0.547. The mean value of relative protein expression of BCL6B in 25 GC tissues was much lower than that in 25 normal gastric mucosal tissues (*p* = 0.038).

### 3.3. Methylation detection of B-cell CLL/lymphoma 6 member B promoter

**Figure 2.**
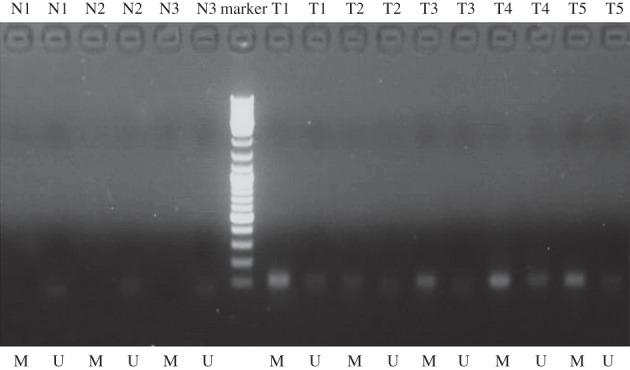
MSP detection of BCL6B promoter methylation in different GC tissues and normal gastric mucosal tissues. T, GC tissues; N, normal gastric mucosal tissues; M, methylated; U, unmethylated.

We detected the different levels of BCL6B promoter methylation (including methylation, non-methylation and partial methylation) in 25 of 459 GC tissues with the MSP analysis, whereas no BCL6B promoter methylation was found in 25 normal gastric mucosal tissues (figure 2). Of 25 GC tissues, five (20%) presented with the methylation of BCL6B promoter, 12 (48%) presented with the partial methylation of BCL6B promoter and eight (32%) presented with the non-methylation of BCL6B promoter.

**Figure 3.**
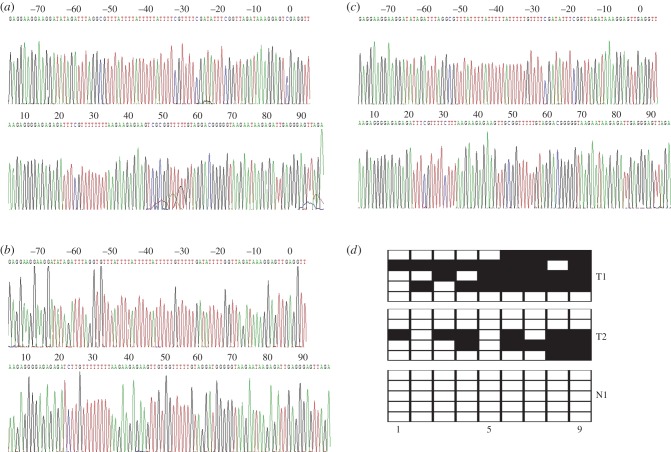
(*a*) Bisulfite sequencing figure of BCL6B in GC tissue 1, (*b*) bisulfite sequencing figure of BCL6B in GC tissue 2, (*c*) bisulfite sequencing figure of BCL6B in normal gastric mucosal tissue and (*d*) bisulfite sequencing results in GC tissues and in normal gastric mucosal tissue. T, GC tissues; N, normal gastric mucosal tissues.

Subsequently, we adopted the bisulfite gene sequencing (BGS)-analysed methylated status of all CpG sites of BCL6B promoter to obtain the precise quantitative methylated degree of BCL6B promoter in all 459 GC patients. Methylated CpG site count of 459 GC patients ranged between 0 and 9. Of the 459 patients included in this study, 381 patients (83.01%) presented one or more methylated CpG sites and 78 patients (16.99%) presented no methylated CpG site. Patients without methylated CpG site had slightly longer mean OS than those with one or more methylated CpG sites (29.40 versus 28.08 months); there is no significant difference between the two groups of patients (*p* = 0.464). According to the result of cut-point analysis for the methylated CpG site count, 239 patients (52.07%) presented three or more methylated CpG sites and 220 patients (47.93%) presented two or fewer methylated CpG sites. No methylated CpG site was found in the normal gastric mucosal epithelial tissues. The methylation sequencing pictures and CpG site charts are shown in figure 3.

### 3.4. Survival analysis

**Figure 4.**
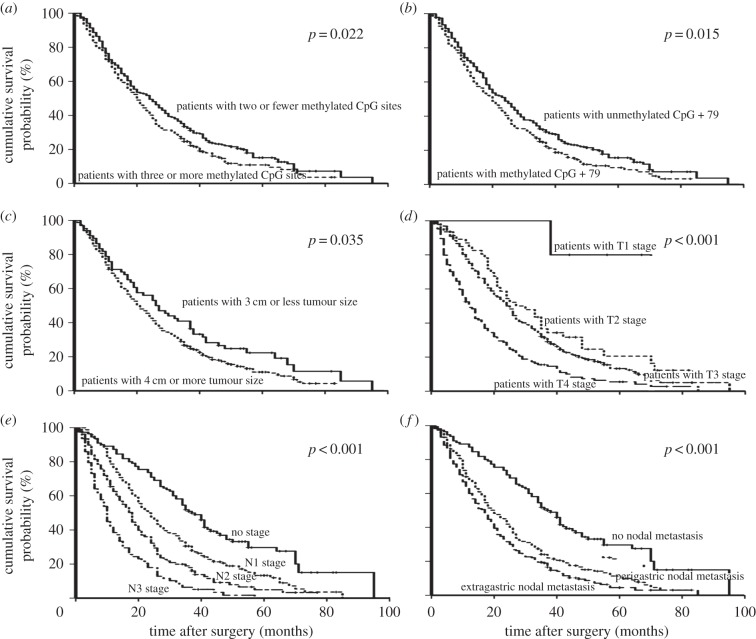
Kaplan–Meier survival curves comparing months of survival in GC patients are shown for (*a*) methylated CpG site count of BCL6B promoter, (*b*) methylated status of CpG +79 of BCL6B promoter, (*c*) tumour size, (*d*) depth of tumour invasion (T stage), (*e*) number of metastatic lymph nodes and (*f*) location of lymph node metastasis.

With the univariate survival analysis, four clinicopathological characteristics were found to have statistically significant associations with OS of 459 GC patients. They were as follows: T stage (*p* < 0.001), N stage (*p* < 0.001), tumour size (*p* = 0.035) and location of lymph node metastasis (*p* < 0.001) (table 2). We also demonstrated that the methylated status of CpG +79 of BCL6B promoter had significant association with the survival of 459 GC patients (*p* = 0.015). In addition, we found that patients with two or fewer methylated CpG sites had much longer median OS than those presenting with three or more methylated CpG sites of BCL6B promoter (*p* = 0.022) (table 2 and figure 4).

**Table 2.** Survival analysis of 459 GC patients in this study.

variables	median OS (mo)	*χ*^2^-value	univariate *p-*value	HR value	multivariate *p-*value	AIC value
gender						
male	22	0.633	0.426			
female	20					
age at surgery (years)						
≤60	20	0.007	0.934			
>60	23					
tumour location						
upper third	21	7.685	0.053			
middle third	18					
lower third	24					
≥2/3 stomach	16					
tumour size (cm)						
<4.0	26	4.429	0.035			
≥4.0	21					
Lauren classification						
intestinal	26	5.576	0.062			
diffuse	20					
mixed	17					
depth of tumour invasion (T stage)						
T1	70	41.108	<0.001	1.510 (1.275–1.788)	<0.001	81.839
T2	27					
T3	24					
T4	12					
number of metastatic lymph nodes (N stage)						
N0	37	101.047	<0.001	1.548 (1.396–1.716)	<0.001	101.178
N1	23					
N2	18					
N3	10					
location of lymph node metastasis						
no	37	49.837	<0.001			
perigastric	20					
extragastric	17					
methylated CpG site count						
2 or less	24	5.218	0.022	1.263 (1.033–1.544)	0.023	78.396
3 or more	20					
methylated status of CpG +79						
unmethylated	24	5.880	0.015			
methylated	20

All of the above six factors were included in a multivariate Cox proportional hazards model with bootstrapping performance to adjust for the effects of covariates. With the multivariate analysis, the independent predictors with the OS of all 459 GC patients were identified to be the methylated CpG site count (HR = 1.263, *p* = 0.023), N stage (HR = 1.548, *p* < 0.001) and T stage (HR = 1.510, *p* < 0.001) (table 2).

Lastly, we demonstrated that the methylated CpG site count of BCL6B promoter had a smaller AIC value than any of the other independent predictors calculated within the Cox proportional hazards regression model, representing the optimal prognostic predictor of GC (table 2).

## 4. Discussion

The human proto-oncogene BCL6 has been identified from chromosomal breakpoints in B-cell lymphomas. It functions as a sequence-specific transcriptional repressor owing to the NH2-terminal half of the BCL6 protein containing a repressor domain [9–12]. The BCL6 gene encodes a 92 to 98 kDa nuclear phosphoprotein [13,14] that contains the BTB/POZ domain in the NH2-terminal region and six Krüppel-type zinc finger motifs in the COOH-terminal region [9–11,15]. BCL6B, a homologue of BCL6, was identified to possess the 94% identical zinc finger motifs of BCL6 [16]. Several authors have reported relevant information for evaluation of the role of BCL6B in malignant disease. BCL6B was identified to be capable of repressing some STAT-induced transcription by binding to DNA sequences recognized by STAT factors [17]. It is believed that the JAK/STAT pathway plays a crucial role in carcinogenesis of GC [18], and repression of activation of STAT factors is a promising method for inhibition of the progress of GC [19]. Angiogenesis, based on the expression of angiogenic factors, is another key component of stomach canceration, and was demonstrated to significantly affect the progress, metastasis and prognosis of GC [20]. Recently, investigators reported that BCL6B, induced by vascular endothelial growth factor (VEGF)-A, supported angiogenic sprouting via BCL6B-CUL3-based poly-ubiquitination-dependent degradation of CBF1 to downregulate Notch signalling [21]. Subsequently, VEGF-A was reported to mediate BCL6B mRNA stabilization by activating heat shock protein 90β [22].

In 2011, BCL6B was demonstrated to be silenced or downregulated in all nine GC cell lines and readily expressed in normal gastric tissues [7]. Loss of BCL6B expression was regulated by promoter hypermethylation. Researchers demonstrated that re-expression of BCL6B in GC cell lines could inhibit colony formation, suppress cell viability, induce apoptosis and restrain tumorigenecity in nude mice [7]. They found that BCL6B hypermethylation was detected, respectively, in 49.0% (102/208) and 66.3% (67/101) of two independent cohorts of patients with GC, and demonstrated that the methylation of BCL6B promoter in GC tissues was an independent factor for the survival of patients by multivariate analysis [7]. Meanwhile, a similar conclusion was also drawn from plasmas of GC patients [8]. Therefore, the importance of BCL6B as a potential tumour suppressor in GC should be highlighted.

In this study, we initially found that 68% (17/25) GC tissues presented BCL6B promoter methylation by using MSP detection. Of those patients with BCL6B promoter methylation, we found that 12 presenting with the partial methylation of BCL6B promoter were detected by the MSP. However, we also found that the methylated degree of 12 patients presenting with the partial methylation of BCL6B promoter were inconsistent (figure 2). Therefore, we thought the detailed information of BCL6B promoter methylation should be quantitatively detected. Actually, many researchers proposed that methylated CpG site detection was appropriate for precise quantitative evaluation of the correlation between methylated levels of gene promoter and various abnormally biological events [23–26]. We meticulously analysed the methylated CpG sites of BCL6B promoter in 459 GC patients by using the bisulfite genomic sequencing (BGS) method with no fewer than five clones for each GC sample. After the methylated CpG site detection of BCL6B DNA promoter, 381 patients (83.01%) presented with one or more methylated CpG sites, and 78 patients (16.99%) presented without a methylated CpG site. However, the optimal cut-off value of the methylated CpG site count of BCL6B promoter was two, calculated by the cut-point survival analysis in 459 GC patients. Of 459 GC patients, 239 (52.07%) presenting with three or more methylated CpG sites had significantly shorter median OS than 220 (47.93%) with two or fewer methylated CpG sites of BCL6B promoter. Although we found that the methylated status of CpG +79 of BCL6B promoter, the methylated CpG site count of BCL6B promoter and other four clinicopathological variables were significantly associated with survival of GC patients, the methylated CpG site count of BCL6B promoter, N and T stages were identified to be the independent predictors of the OS of 459 GC patients by Cox regression with forward step procedures. Owing to its smaller AIC value, the methylated CpG site count of BCL6B promoter was ultimately demonstrated to be the optimal predictor of GC patients' prognosis by using the AIC value calculation within the Cox regression. The findings in this study indicate that quantitative BGS detection of BCL6B promoter methylation is appropriate to elucidate the methylated levels of BCL6B that can be applicable for accurate survival analysis.

## 5. Patients and methods

### 5.1. Data source

After the institutional review board of Tianjin Medical University Cancer Hospital (China) approved our study, data from the cancer registry of the Tianjin Cancer Institute were obtained. Data obtained from the registry were listed as follows: age, gender, tumour location, tumour size, depth of tumour invasion (T stage, according to the UICC TNM Classification for GC, 6th edn), number of metastatic lymph nodes (N stage, according to the UICC TNM Classification for GC, 6th edn), extent of lymph node metastasis, Lauren classification and follow-up vital status. Oral and written informed consents were also obtained from patients who were included in this study.

### 5.2. Patients and study samples

To analyse BCL6B promoter methylation, we collected 459 fresh GC tissues from patients with GC who underwent curative gastrectomy between April 2003 and December 2007 at the Department of Gastroenterology, Tianjin Medical University Cancer Hospital. A cohort of 25 normal gastric mucosal epithelial tissues from normal people was also obtained between 2004 and 2007 at the Department of Endoscopic Examination and Treatment, Tianjin Medical University Cancer Hospital. The tumour and normal gastric mucosal epithelial tissue samples were histologically verified. The patients were not subjected to radiation, chemical or biological treatment before potentially curative gastrectomy was performed. Adjuvant chemotherapy or radiotherapy was not routinely administered to the patients. The clinicopathological characteristics of these 459 GC patients are summarized in table 1. Consent regarding the use of tissue samples and records was obtained from each patient.

### 5.3. Surgical treatment

Curative resection was defined as the complete absence of grossly visible tumour tissues and metastatic lymph nodes remaining after resection was performed with pathologically negative resection margins. Primary tumours were resected en bloc with limited or extended lymphadenectomy (D1 or D2–3 according to the Japanese Gastric Cancer Association). Surgical specimens were evaluated according to the UICC TNM Classification for GC (6th edn).

### 5.4. DNA and RNA extractions

Genomic DNA was extracted from the 459 GC tissues and the 25 normal gastric mucosal tissues by using a QIAamp DNA mini kit (Qiagen, Valencia, CA) according to the manufacturer's instructions. Genomic DNA was modified using sodium bisulfite in EZ DNA Methylation-Gold kit (Zymo Research, Hornby, Canada). RNA was extracted from 25 of the 459 GC tissues and 25 normal gastric mucosal tissues by using Trizol reagent (Invitrogen, Carlsbad, CA) according to the manufacturer's instructions.

### 5.5. Western blotting analysis

A total of 25 of the 459 GC tissues and 25 normal gastric mucosal tissues were each added to 1 ml of 100 mmol l^−1^ Tris/HCl (pH 7.5), 100 mmol l^−1^ NaCl, 0.5% sodium deoxycholate, 1 mmol l^−1^ ethylenediaminetetraacetic acid, 1% Nonidet P-40, 0.1% sodium dodecyl sulfate and protease inhibitor. After blocking was performed, 50 µg of the sample was incubated for 60 min with mouse anti-BCL6B (Santa, sc56625, 1 : 1000 dilution) at room temperature. A gel imager system (Asia Xingtai Mechanical and Electrical Equipment Company, Beijing, China) was used to analyse images and determine grey values.

### 5.6. Semi-quantitative RT-PCR analysis

The mRNA expression of BCL6B was detected by subjecting 25 of the 459 GC tissues and 25 normal gastric mucosal tissues to RT-PCR. Total RNA was reverse-transcribed to cDNA in a 20 µl solution by using a reverse transcription kit (Invitrogen). The primers designed and used for BCL6B were listed as follows: forward sequence, 5′-TTGCTGTAGTTTGGTTGGGATT-3′; reverse sequence, 5′-ATGGGGAGAAAGAGGGAAGAG-3′. The GAPDH gene was used as an endogenous control of semi-quantitative DNA-PCR. Primers designed and used for GAPDH were listed as follows: forward sequence, 5′-GAAGGTGAAGGTCGGAGTC-3′; reverse sequence, 5′-GAAGATG GTGATGGGATTTC-3′. The following PCR cycling conditions were applied: 35 cycles of denaturation at 95°C for 3 min, annealing at 94°C for 30 s and extension at 56°C for 30 s, and a final extension at 72°C for 8 min. PCR products were electrophoresed on 2% agarose gel with ethidium bromide and visualized using a gel imager system (Asia Xingtai Mechanical and Electrical Equipment Company, Beijing, China).

### 5.7. Sodium bisulfite treatment

The genomic DNA was modified using sodium bisulfite in an EZ DNA Methylation-Gold kit.

### 5.8. Methylation-specific PCR

Twenty-five of 459 GC tissues and 25 normal gastric mucosal tissues were subjected to qualitative methylation analysis of the BCL6B promoter by methylation-specific PCR (MSP). The following BCL6B primers were used to detect the methylated (M) or unmethylated (U) alleles of the BCL6B promoter: for methylated alleles, BCL6B-MF, 5′-TTTTTATTTTCGTTTTCGATATTTC-3′ and BCL6B-MR, 5′-CGTCCTACAAAAACCGCG-3′; for unmethylated alleles, BCL6B-UF, 5′-TTATTTTTATTTTTGTTTTTGATATTTT-3′ and BCL6B-UR, 5′-CCCATCCTACAAAAACCACA-3′. A total of 25 cycles of MSP were performed using Ampli Taq-Gold (methylation-specific primers, annealing temperature 600°C; unmethylation-specific primers, annealing temperature 580°C). MSP primers were initially evaluated to verify whether or not any unbisulfited DNA is amplified, and the specificity of MSP was further confirmed by directly sequencing some PCR products. PCR was resolved using 2% agarose gel.

### 5.9. Bisulfite genomic sequencing

The methylation of the BCL6B promoter in 459 GC tissues and 25 normal gastric mucosal tissues was qualitatively analysed by the BGS. Hot start PCR with bisulfite-treated DNA was performed using a 173 bp PCR product spanning the promoter region from –78 bp to +95 bp relative to the transcription start site of BCL6B. The promoter region of BCL6B contains 11 CpG sites. The sequences of the PCR primers were listed as follows: forward sequence, 5′-GAGGAAGGAAGGATATAGATTTAGG-3′; reverse sequence, 5′-TCTAACTCCCTCAATCTCTTATTCTTAC-3′. The purified PCR products were cloned into the pUC18-T vector (Biodee, Beijing, China), and at least five clones from each sample were randomly selected and sequenced by Shanghai Sangon Co. (Shanghai, China).

### 5.10. Follow-up

After curative surgery, all of the patients were followed up every three months or six months for 2 years at the outpatient department; these patients were also followed up every year from the third year to the fifth year. Thereafter, these patients were followed up annually until their death. The median follow-up of the entire cohort was 44 months, ranging from 2 to 104 months. The follow-up of the patients included in this study was completed in December 2012. Ultrasonography, CT scans, chest X-ray and endoscopy were performed at every visit.

### 5.11. Statistical analysis

Median OS was determined using Kaplan–Meier method, and log-rank test was performed to determine significance. Potentially important factors in univariate analyses (*p* < 0.05) were included in multivariate analyses. OS was subjected to multivariate analysis by using the Cox proportional hazard model with forward step procedures. Hazard ratio (HR) and 95% confidence interval were calculated. Akaike information criterion (AIC) in a Cox proportional hazard regression model was calculated in terms of different categories to quantify their discriminatory ability. A small AIC value corresponds to an efficient model to predict outcomes [27]. With cut-point survival analysis [28], the optimal cut-off of the CpG site count was 2. Significance was set at *p* < 0.05. Statistical analyses were performed using SPSS 18.0.

## Notes

The Institutional Research Ethics Committee of Tianjin Medical University Cancer Hospital approved the study protocol and provided permission to use patient clinical data.
